# Association of Low Birth Weight with Bone Mineral Density and Fracture Occurrence in Postmenopausal Women

**DOI:** 10.1007/s00223-026-01642-3

**Published:** 2026-11-11

**Authors:** Ilari Kuitunen, Ulla Sankilampi, Essi-Mari Manninen, Heikki Kröger, Reijo Sund

**Affiliations:** 1 Department of Pediatrics, Kuopio University Hospital, Kuopio, Finland; 2 Institute of Clinical Medicine, University of Eastern Finland, Kuopio, Finland; 3 Department of Orthopaedics and Traumatology, Kuopio University Hospital, Kuopio, Finland; 4 Kuopio Musculoskeletal Research Unit, University of Eastern Finland, Kuopio, Finland; 5 Clinical Research Center, Kuopio University Hospital, Kuopio, Finland; 6 Neonatal Department, Kuopio University Hospital, Puijonlaaksontie 2, Kuopio, 70211 Finland

**Keywords:** Fracture, Epidemiology, Preterm birth

## Abstract

Low birth weight (LBW) has been associated with impaired skeletal development and low peak bone mass. However, the association between LBW and fracture risk in postmenopausal women remains un-clear. We analyzed prospectively collected data from the population-based Osteoporosis Risk Factor and Pre-vention (OSTPRE) cohort, including women aged >50 years. Birth weight was categorized as LBW (<2500 g) or normal birth weight (≥2500 g). Fracture outcomes were analyzed using standardized inci-dence ratios (SIRs) with 95% confidence intervals (CIs). Bone mineral density (BMD) trajectories were assessed using linear mixed-effects models. There were 635 women in the LBW group and 9387 in the normal birth weight group. During follow-up, a total of 8453 fractures occurred. Women with LBW had an 18% higher risk of vertebral fractures (SIR 1.18, 95% CI 1.01–1.39), a 20% higher risk of vertebral or hip fractures combined (SIR 1.20, 95% CI 1.06–1.38), and an 11% higher risk of any osteoporotic fracture (SIR 1.11, 95% CI 1.01–1.22) compared with women with normal birth weight. Hip fracture risk was borderline increased (SIR 1.25, 95% CI 0.99–1.59). No significant differences in BMD levels or age-related BMD decline were observed between the groups. LBW was associated with increased fracture risk in postmenopausal women despite similar BMD trajecto-ries. These findings suggest that adverse early-life conditions may have long-term effects on skeletal fra-gility independent of later-life BMD.

**Supplementary Information:**

The online version contains supplementary material available at https://doi.org/10.1007/s00223-026-01642-3.

## Introduction

Preterm birth and low birth weight are associated with several health problems in both the short and long term [1]. The third trimester of pregnancy which is truncated by preterm birth, is a critical period for bone mineralization, although mineralization continues throughout infancy and childhood [2]. Low Birth Weight (LBW) is tightly correlated with prematurity, and studies have suggested that both LBW and preterm birth may lead to a persistent reduction in peak bone mass, and lower bone mineral density [2–4].

Previous evidence suggests that LBW may be associated with an earlier onset of natural menopause, although findings have been inconsistent [5, 6]. A non-linear association has been reported, with women born weighing < 2500 g reaching natural menopause at a median age of 51 years, compared with 52 years among those weighing 3500–3999 g at birth [5]. Earlier age at menopause has, in turn, been associated with an increased risk of fractures and osteoporosis [7, 8]. Together, these findings raise the possibility that LBW may contribute to greater fracture susceptibility later in life through its association with earlier menopause. More broadly, the influence of early-life factors on reproductive aging, combined with the well-established relationship between menopause timing and bone health, suggests that adverse early developmental conditions such as LBW could have long-term consequences for skeletal health after menopause. However, this potential pathway has not been previously investigated.

Thus, we aimed to investigate the association between birth weight, BMD, and fracture risk in postmenopausal women aged over 50 years. Our hypothesis was that low birth weight is associated with a higher risk of fractures and lower bone mineral density after menopause.

## Methods

The study utilizes data from the Osteoporosis Risk Factor and Prevention study (OSTPRE) that is a large population-based, prospective cohort study, launched in 1989, capturing all (*n* = 14,220) women born 1932–41 residing in Kuopio Province, Eastern Finland. Information on the participants’ health, sociodemographic factors and lifestyle has been collected at 5-year intervals [9].

### Birth Weight Classification

Birth weight has been acquired in the OSTPRE dataset from the 2.5-year follow-up questionnaire in 1991 and the 15-year follow-up questionnaire in 2004. Birth weight was asked by either reporting the exact weight or selecting a weight category (underweight, normal weight, heavier than normal, or “I don’t know”). The categories were not defined using numerical cut-offs in the questionnaire. Additionally, participants were asked whether they had a written certificate of birth weight. A total of 12 625 people responded, with 3553 providing an exact weight. There were 9387 individuals in normal weight category, 635 in low-birth-weight category and 2603 in unknown category. The exact birth weight is prioritized in the analysis if both the birth weight and the weight category have been reported. Numerical birth weight values were checked, and inconsistencies were corrected as follows. Primarily, written certificates were relied upon and used as such. If the weight was < 100 g, this was interpreted as a unit error or otherwise incorrect. If a numerical weight was available in the 2.5-year follow-up questionnaire, it was used. If numerical weight was available in both questionnaires but differed, their mean was used. LBW was defined as birth weight below 2500 g. Women reporting an exact birth weight were classified as LBW or normal birth weight using this cut-off, and women reporting only a category were classified according to that category. Women in the unknown category were excluded from the analyses.

### Fractures

Fractures were identified by using self-reported fractures in surveys that were validated from the medical records as well as from the register data (Finnish Hospital Discharge Register, the Care Register for Health Care, and the Register of Primary Health Care Visits) using diagnosis codes [10]. We also utilized radiologic information and imaging services data for the identification of wrist and proximal humerus fracture [11, 12]. Participants reported the fracture site, date and treating healthcare provider in free text. All reported fractures were verified from the medical records of the treating provider or from the registers, incorrectly reported sites were corrected, and only verified fractures were included [13]. We focused on the five common fracture types: hip (ICD-10: S72.0–2), forearm (ICD-10: S52), humerus (ICD-10: S42.2–4), vertebral (ICD-10: S12, S22.0–1, S32.0–2, S32.7–8, M80, M48.4–5, T08, T91.1), and ankle fractures (ICD-10: S82.3–8). A wash-out period of 1 year was used to separate repeated fractures of the same type unless we had more detailed data from manual validations. All but ankle fractures were considered as osteoporotic fractures.

### Bone Mineral Density

Femoral neck bone mineral density measurements were obtained from the DXA scans performed each fifth year as a part of OSTPRE follow-up visits and additionally from clinical measurements conducted at the Kuopio University Hospital between 2007 and 2022. The research DXA group was a stratified sample of 3,220 women selected among the volunteers who responded to the baseline questionnaire in 1989 (approximately 2,500 randomly selected and 700 from risk groups), and they were measured at 5-year intervals from 1989 to 2019. Clinical measurements were performed when clinically indicated. Four types of instruments were used: a Lunar DPX scanner (Lunar, Madison, WI, USA), a DPX-IQ scanner, GE Healthcare Lunar Prodigy, and iDXA Dual-Energy X-Ray Densitometer. The femoral neck T-score value was determined according to NHANES III reference population of white women aged 20 to 29 years [14]. The research measurements were standardized across scanners as described previously [14].

### Statistical Methods

Fractures were examined using Poisson regression to calculate standardized incidence ratios (SIR) with 95% confidence intervals (CI). The SIRs were standardized for age. Adult height was not adjusted for, as LBW is associated with shorter adult height and height may therefore lie on the pathway between birth weight and fracture risk. In the analyses, LBW group was compared to normal birth weight group. Fractures were classified into hip, vertebral, hip or vertebral, forearm, humerus, ankle, and any osteoporotic fractures. Follow-up was from the age of 50 (earliest at year 1983) until the age of 90 (or the final day of the year 2024). Women with missing data were excluded, and the analyses were based on complete cases.

Bone mineral density (femoral neck T-scores) association with age was visualized using LOESS smoothed trends and LBW group was compared to normal birthweight group using a linear mixed effects model taking repeated BMD measures into account [15].

### Ethics and Permissions

The OSTPRE study has received ethical approval from the Kuopio University Hospital Ethics Committee on October 28, 1986. All participants provided written informed consent prior participation to the study. Additional register data permissions were obtained from the Social and Health Data Permit Authority Findata (THL/6840/14.02.00/2020, THL/5581/14.06.00/2025).

## Results

Of the 14,220 women born 1932–41 and included in the OSTPRE study, 12 625 people responded overall to birthweight question. There were 9387 individuals in normal weight category, 635 in low-birth-weight category and 2603 in unknown category. (Figure S1). A total of 3553 participants provided an exact birthweight. Most birthweight observations were concentrated in the range of 3000–4000 g (Figure S2). There were totally 8453 (1081 hip, 2474 vertebral, 2754 forearm, 926 humerus, and 1218 ankle) fractures during 345,300 person years of follow-up in LBW and normal groups.

The risk of vertebral fractures was 18% higher (SIR 1.18, CI 1.01–1.39), vertebral or hip fractures was 20% higher (SIR 1.20, CI 1.06–1.38), and any osteoporotic fracture was 11% higher (SIR 1.11, CI 1.01–1.22) in LBW group compared to normal birth weight. (Table 1) The risk of hip fractures was borderline increased in the LBW group (SIR 1.25, CI 0.99–1.59). Risks for forearm, humerus, and ankle fractures showed no evidence of difference between LBW and normal birth weight groups. (Table 1)

**Table 1:** Comparison of fractures between normal and low birth weight groups

Outcome	SIR	95% CI
Hip fracture	1.25	0.99–1.59
Vertebral fracture	1.18	1.01–1.39
Vertebral or hip fracture	1.20	1.06–1.38
Forearm fracture	1.03	0.88–1.19
Humerus fracture	1.01	0.77–1.32
Ankle fracture	1.03	0.82–1.29
Any osteoporotic fracture	1.11	1.01–1.22

Standardized incidence ratio (SIR) with 95% confidence intervals used in the analysis and low birth weight group (*n* = 635) compared to normal birthweight (*n* = 9387). Any osteoporotic fracture includes hip, vertebral, forearm and humerus fractures

**Fig. 1:**
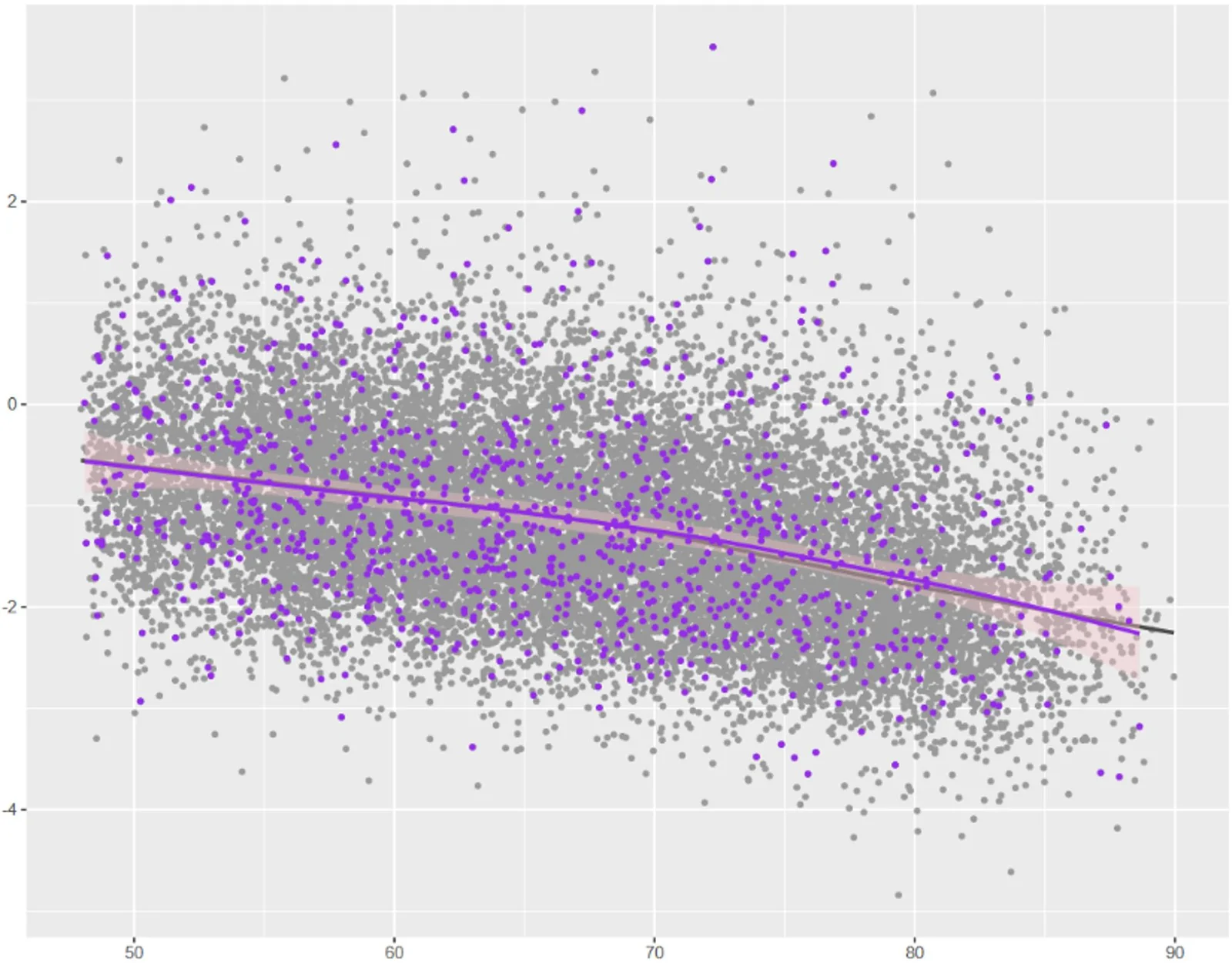
Bone mineral density measurements by age, with low-birth-weight individuals (purple) compared to women with normal birth weight (grey); the curves represent group means. Age (years) is shown on the x-axis and femoral neck T-score (SD units) on the y-axis

There were 1028 BMD T-scores for 270 women in the LBW group and 14416 T-scores for 3844 women in the normal group. Mean BMD decreased by age (-0.0323 T-scores by one year of age, CI -0.0345 to -0.0300) but LBW and normal groups were similar in terms of trends (-0.0319 T-scores, CI -0.0361 to -0.0276) in LBW vs. -0.0327 (CI -0.0338 to -0.0315) in normal group) and mean levels at age of 65 (T-score − 1.13, CI -1.23 to -1.03) in LBW vs. -1.17 (CI -1.20 to -1.14) in normal group). (Fig. 1)

## Discussion

In this population-based cohort study, low birth weight was associated with an increased risk of fractures in postmenopausal women aged over 50 years. The association was particularly evident for vertebral and hip fractures, which are among the most clinically relevant osteoporotic fractures. To our knowledge, evidence on birth weight and fracture risk specifically in postmenopausal women is extremely limited, as previous studies have mainly focused on childhood fracture outcomes or adult bone mineral density rather than fractures themselves.

Interest in the long-term effects of early-life factors on adult skeletal health has increased alongside the Developmental Origins of Health and Disease hypothesis, which proposes that adverse fetal and early postnatal exposures may influence disease susceptibility later in life [16, 17]. In the context of bone health, low birth weight and prematurity have been associated with impaired skeletal mineralization and lower peak bone mass [2–4]. However, previous findings regarding adult bone mineral density have been inconsistent [18, 19], and most earlier studies have included younger populations (age less than 30 years) in whom the effects of aging and menopause on skeletal fragility could not yet be fully evaluated.

An important finding of our study was that fracture risk differed between birth weight groups despite largely similar bone mineral density trajectories. This could mean that mechanisms beyond bone mineral density contribute to fracture susceptibility among individuals with low birth weight. Potential explanations include differences in bone quality or microarchitecture, muscle mass, frailty, falls, menopause timing, or lifelong physical activity patterns.

Two previous Finnish studies found fewer fractures among individuals born preterm and LBW compared with those born at term, particularly among boys [20, 21]. These observations appear conflicting given the evidence linking LBW with lower adult bone mineral density [2]. It has been suggested that behavioural and lifestyle factors, such as lower participation in physical activity or reduced risk-taking behaviour, may partly explain these findings [2, 20]. In contrast, our results indicate that fracture susceptibility may emerge later in life, when age-related bone loss and postmenopausal skeletal fragility become increasingly important.

Our findings differ from those of Byberg et al., who found no association between birth weight and fracture risk at ages 50 to 94 years in two Swedish cohorts with birth weight obtained from birth records [22]. Their cohorts included both men and women born in 1915 to 1929, and birth weight was analysed as a continuous exposure. We studied women only and compared LBW with normal birth weight. Differences in exposure ascertainment and fracture identification may also contribute to the differing findings.

Gestational age was not available, and we could not distinguish whether LBW reflected preterm birth or fetal growth restriction. These conditions may affect the skeleton differently, as preterm birth truncates the period of most rapid fetal bone mineral accretion in the third trimester, whereas growth restriction reflects reduced nutrient supply throughout pregnancy [2, 16]. In the 1930s, survival among very preterm and very low birth weight infants was low, so most surviving women with LBW in our cohort were likely born moderately preterm or growth restricted near term. Our findings should therefore not be directly generalized to adults born very preterm in the era of modern neonatal intensive care.

The major strength of this study is the long-term prospective follow-up of a large population-based cohort extending from birth-related factors to fracture outcomes after menopause. In addition, repeated bone mineral density measurements enabled evaluation of longitudinal skeletal changes over time. However, the study also has limitations. Birth weight data were partly self-reported and may therefore be subject to recall bias or misclassification. Most women reported only a weight category without predefined cut-offs, which may have caused some cases of misclassification. Birth weight was analysed categorically, and dose-response or non-linear associations could not be assessed. Information on gestational age was unavailable, preventing separation of the effects of prematurity and fetal growth restriction. Only clinically diagnosed vertebral fractures were captured, and many vertebral fractures may have remained undiagnosed. This underestimates vertebral fracture incidence, but detection is unlikely to differ by birth weight group, and the relative estimates are therefore unlikely to be biased. Menopausal status was not individually ascertained, and early menopause, hormone therapy and hysterectomy were not considered. The BMD sample included women from risk groups and clinical measurements after 2007, which may have been performed in women with lower BMD than average, whereas women with poorer health were more likely to drop out of research visits. Different DXA scanners were used during the follow-up. The cohort included only women who survived to adulthood and lived in the region in 1989, and selective early-life mortality among LBW infants may have attenuated the associations. These women were born just before or during the Second World War, and wartime food shortages may have affected early skeletal development in both groups. Furthermore, the SIRs were standardized only for age, and residual confounding related to lifestyle factors, physical activity, nutrition, and socioeconomic status cannot be excluded.

## Conclusion

Low birth weight was associated with increased fracture risk in postmenopausal women despite similar bone mineral density trajectories. These findings suggest that adverse early-life conditions may have long-term effects on skeletal fragility that are not fully explained by bone mineral density alone. Further studies are needed to clarify the biological mechanisms underlying this association.

## Supplementary Information

Below is the link to the electronic supplementary material.

Supplementary file 1.

## Data Availability

No datasets were generated or analysed during the current study.
