# Supplementary material for: Association of Low Birth Weight with Bone Mineral Density and Fracture Occurrence in Postmenopausal Women
Source: Calcif Tissue Int. 2026 Nov 11;117(1):179. doi: 10.1007/s00223-026-01642-3 (PMC13751831; doi:10.1007/s00223-026-01642-3)
Supplement: Supplementary file 1 [file 223_2026_1642_MOESM1_ESM.docx]

**Supplementary materials:**

**Figure S1** Flow diagram of participant selection to study.

**Figure S2** Birth weight density plot. Birth weight (g) on the x-axis and density (probability per gram) on the y-axis.
